# Single‐Cell and Bulk Transcriptomics Identify GNGT1‐High Malignant Epithelial Cells Associated With Immune Suppression in Esophageal Squamous Cell Carcinoma

**DOI:** 10.1111/cbdd.70381

**Published:** 2026-08-12

**Authors:** Qian Yuan, Cheng Wang, Yintao Chang, Qiang Lyu, Yuxiang Jin, Lei Xue

**Affiliations:** ^1^ Department of Thoracic Surgery Second Affiliated Hospital of Naval Medical University Shanghai China

**Keywords:** biomarker, esophageal squamous cell carcinoma, GNGT1, immune microenvironment, machine learning, single‐cell transcriptomics

## Abstract

Esophageal squamous cell carcinoma (ESCC) features epithelial heterogeneity and an immunosuppressive microenvironment, yet clinically relevant malignant epithelial states remain poorly defined. We integrated four single‐cell RNA‐sequencing datasets and 10 bulk transcriptomic cohorts totaling 1318 samples, and applied cross‐cohort differential expression, survival analysis, and a machine‐learning framework of 113 algorithm combinations to screen for malignant epithelial cell‐associated biomarkers. GNGT1 was prioritized for its consistent upregulation, prognostic association, and limited prior characterization in ESCC. Across independent cohorts, GNGT1 exhibited favorable diagnostic performance, while high expression correlated with poorer overall survival and more advanced local tumor status. Immune deconvolution consistently linked GNGT1‐high tumors to reduced CD8^+^ T‐cell infiltration, lower immune scores, and decreased cytotoxic T‐cell markers. Single‐cell analyses further associated GNGT1‐high epithelial cells with altered epithelial–immune communication involving MIF, prostaglandin, and CXCL signaling, alongside enrichment of epithelial–mesenchymal transition, mTORC1, and proliferative programs; qPCR confirmed elevated GNGT1 expression in ESCC cell lines. This study defines GNGT1 as a marker of an immunosuppressive malignant epithelial state, thereby bridging epithelial heterogeneity with immune remodeling in ESCC. These findings support GNGT1 as a candidate diagnostic, prognostic, and biologically informative biomarker warranting mechanistic and clinical validation.

## Graphical abstract

This study integrates single‐cell and bulk transcriptomes with machine learning to identify GNGT1 as an ESCC biomarker. Pan‐cancer, immune infiltration, pathway, and cell–cell communication analyses reveal TME associations. Exploratory treatment cohorts, in silico drug sensitivity, and molecular docking further support its therapeutic potential.

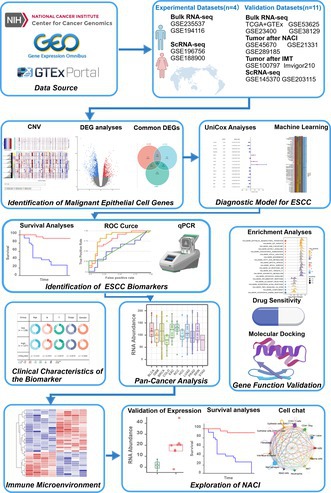

**Abbreviations**
AUC: area under the curve
BP: biological process
CC: cellular component
CNV: copy number variation
CTRP: cancer therapeutics response portal
DEGs: differentially expressed genes
EMT: epithelial‐mesenchymal transition
ESCC: esophageal squamous cell carcinoma
FC: fold change
fgsea: fast gene set enrichment analysis
GDSC: genomics of drug sensitivity in cancer
GEO: gene expression omnibus
GNGT1: G protein subunit gamma T1
GO: gene ontology
IC50: half‐maximal inhibitory concentration
KEGG: kyoto encyclopedia of genes and genomes
KM: Kaplan–Meier
LASSO: least absolute shrinkage and selection operator
MF: molecular function
MIF: macrophage migration inhibitory factor
PCA: principal component analysis
PDB: Protein Data Bank
qRT‐PCR: real‐time quantitative PCR
RF: random forest
ROC: receiver operating characteristic
scRNA‐seq: single‐cell RNA sequencing
SVM: support vector machine
TCGA: the Cancer Genome Atlas
TME: tumor microenvironment
UCell: single‐cell pathway activity scoring R package
UMAP: uniform manifold approximation and projection

## 1 Introduction

Esophageal squamous cell carcinoma (ESCC) is a common digestive tract malignancy, with over 500,000 new cases annually, nearly half occurring in China (Morgan et al. 2022). Despite advances in surgery, radiotherapy, chemotherapy, and multimodal treatment, the 5‐year overall survival remains below 30%, largely because of delayed diagnosis, frequent recurrence or metastasis, and resistance to existing therapies (Bray et al. 2024; Jiang et al. 2025). Thus, better understanding of ESCC molecular features and identification of clinically relevant biomarkers are critical for risk stratification and treatment decisions.

The tumor microenvironment (TME) is closely implicated in ESCC progression and treatment response (Zhang et al. 2021). Malignant epithelial cells interact with immune and stromal cells through complex intercellular networks, shaping tumor growth, immune escape, and resistance (Chang et al. 2025). Single‐cell RNA‐sequencing (scRNA‐seq) enables high‐resolution dissection of ESCC heterogeneity (Jia et al. 2023). However, malignant epithelial cell states and their associated biomarkers remain incompletely characterized, particularly in the context of epithelial‐immune interactions. Machine‐learning‐based approaches have increasingly been used to assist biomarker screening (Qin et al. 2024). Integrating single‐cell transcriptomics, bulk transcriptomic cohorts, and machine‐learning‐assisted prioritization may help identify biologically meaningful biomarkers associated with malignant epithelial cell programs in ESCC.

G protein signaling is involved in multiple cancer‐related processes, including cell proliferation, migration, survival, and microenvironmental remodeling (Wang et al. 2018). Heterotrimeric G proteins consist of α, β, and γ subunits; the γ subunits mediate transmembrane signal transduction and downstream cascades (Chen et al. 2007). G protein subunit gamma transducin 1 (*GNGT1*) is predominantly expressed in retinal photoreceptors and mediates visual signal transduction under physiological conditions (Cheng et al. 2023). Recent studies have reported aberrant *GNGT1* expression in lung adenocarcinoma, gastric cancer, and other malignancies (Fan et al. 2025; Huang et al. 2025). However, its expression pattern, clinical relevance, and role in the ESCC TME remain poorly understood.

In this study, we integrated multiple single‐cell and bulk transcriptomic datasets to identify malignant epithelial cell‐associated biomarkers in ESCC. Through cross‐cohort differential expression, survival analysis, and machine‐learning‐assisted screening, we prioritized *GNGT1* for further investigation. We evaluated its expression, diagnostic and prognostic value in independent cohorts and by qPCR in ESCC cell lines, and performed immune infiltration, pathway, and single‐cell communication analyses to explore its association with the immune TME. Exploratory therapeutic cohort and in silico drug‐sensitivity analyses were also conducted. Together, these analyses provide insight into a *GNGT1*‐associated malignant epithelial cell state and its relationship with immune remodeling in ESCC.

## 2 Materials and Methods

### 2.1 Data Source and Preprocessing

All scRNA‐seq and bulk transcriptomic datasets used in this study were obtained from publicly available repositories, including the Gene Expression Omnibus (GEO), The Cancer Genome Atlas (TCGA), and the Genotype‐Tissue Expression (GTEx) project. No newly generated RNA‐seq or microarray data are included. A complete list of datasets, including accession numbers, sample sizes, platform information, and respective usage, is provided in Table S1. Detailed preprocessing, quality control, normalization, and batch‐effect correction procedures are described in the Methods S1 (Johnson et al. 2007; Liu et al. 2021; Zhao et al. 2021).

### 2.2 scRNA‐Seq Analysis

scRNA‐seq data were processed with the standard pipeline using Seurat (v4.4.0; Hao et al. 2021). Cell quality control criteria were: gene count 200–5000, mitochondrial gene ratio < 20%, and genes expressed in at least 3 cells. After global normalization, the top 2000 highly variable genes were selected for principal component analysis (PCA), and the top 20 principal components (PCs) were retained. Batch effects were corrected using Harmony (v0.1.1). Cell clusters were identified by graph‐based Louvain clustering (resolution 0.1) and UMAP visualization (Korsunsky et al. 2019). Cell type annotation was performed by integrating CellMarker and signature genes from published ESCC single‐cell studies (Yao et al. 2020). For malignant epithelial cell identification: epithelial subsets were extracted and re‐clustered; inferCNV (v1.18.1) was used to infer copy number variations (CNV) and calculate CNV scores with T cells as diploid references (Patel et al. 2014). Patients were stratified into *GNGT1*‐high and *GNGT1*‐low groups based on the median *GNGT1* expression level. Cell–cell communication networks were constructed using CellChat (v1.6.1), focusing on the signaling hub role and core pathways of *GNGT1*‐high epithelial cells.

### 2.3 Functional and Pathway Enrichment Analysis

Gene Ontology (GO) and Kyoto Encyclopedia of Genes and Genomes (KEGG) enrichment analyses were performed on the 231 malignant epithelial cell‐associated candidate genes using the R package clusterProfiler (v4.8.3; Kanehisa et al. 2023). GO analysis covered three dimensions: biological process (BP), cellular component (CC), and molecular function (MF). The h.all.v2026.1.Hs.symbols signature gene set was obtained from the MSigDB database (https://www.gsea‐msigdb.org/gsea/msigdb). Gene set enrichment analysis (GSEA) was conducted using the R package fgsea (v1.26.0; Wang et al. 2024). Samples were stratified into *GNGT1*‐high and *GNGT1*‐low groups according to the median *GNGT1* expression level. The number of permutations was set to 1000, and significant enrichment was defined as |normalized enrichment score (NES)| ≥ 1.5 and adjusted *p* < 0.05. UCell (v2.6.2) and ssGSEA modules from GSVA (v1.50.5) were used to determine the cell‐type specificity of key pathways (Andreatta and Carmona 2021).

### 2.4 Machine Learning‐Assisted Prioritization of Diagnostic Candidate Biomarkers

To identify malignant epithelial cell‐associated genes with both prognostic and diagnostic relevance, univariate Cox regression was first performed, and 13 prognosis‐associated candidate genes were retained for subsequent analysis. A machine‐learning‐assisted screening framework comprising 113 algorithm combinations derived from 12 basic algorithms was then applied to further prioritize candidate biomarkers. The 12 base algorithms were selected to represent diverse methodological families, including: (1) regularized regression methods (LASSO, Ridge, and ElasticNet), which perform feature selection through L1/L2 regularization and are well‐suited for high‐dimensional data; (2) generalized linear model‐based methods (Stepglm, glmBoost, and plsRglm), which provide interpretable probabilistic outputs; (3) ensemble learning methods (Random Forest, GBM, and XGBoost), which capture non‐linear relationships and improve generalization through bootstrap aggregation and boosting; and (4) pattern‐recognition methods (SVM, LDA, and naive Bayes), which are effective for classification tasks with different underlying data distributions. The inclusion of algorithms from different families ensures that the screening process is not biased toward a single modeling assumption and leverages the complementary strengths of diverse approaches.

GSE53625 was used as the training cohort, and 10‐fold cross‐validation was applied during model training to reduce overfitting. For each algorithm combination, hyperparameters were optimized via grid search within the cross‐validation loop. TCGA‐ESCC, GSE23400, and GSE38129 were used as independent validation cohorts to evaluate the discriminatory performance of the candidate gene signatures. ROC curves were generated using the pROC package (v1.18.5), and AUC values were calculated to assess diagnostic relevance. Algorithm combinations were ranked according to the mean AUC across the training and validation cohorts, and genes retained by the best‐performing model were selected for further biological and clinical evaluation. All machine learning analyses were completed on the BioBean online analysis platform (http://www.sxdyc.com/; Chan et al. 2023). The methods for pan‐cancer analysis of *GNGT1* are described in Methods S2.

### 2.5 Tumor Immune Microenvironment Infiltration Analysis

ESCC patients were stratified into *GNGT1*‐high and *GNGT1*‐low groups according to the median *GNGT1* expression level. Immune cell infiltration was estimated using seven algorithms, including TIMER, MCP‐counter, CIBERSORT, CIBERSORT‐ABS, EPIC, quanTIseq, and xCELL (Yang et al. 2023). Differences in immune cell abundance between groups were compared using the Wilcoxon rank‐sum test. Spearman correlation analysis was used to assess the associations of *GNGT1* expression with immune checkpoint molecules and CD8^+^ T cell marker genes.

### 2.6 Quantitative Real‐Time PCR (qRT‐PCR)

Total RNA was extracted from HET‐1A, TE1, TE13, and KYSE150 cells using TRIzol reagent (Life Technologies, USA). Reverse transcription was performed using 5× HiScript II qRT SuperMix (Vazyme, Nanjing, China). qRT‐PCR was carried out using SYBR Green Premix (Vazyme) on a QuantStudio 5 Real‐Time PCR System (Applied Biosystems, USA) with a three‐step amplification protocol. All samples were run in technical triplicates. *GNGT1* expression was normalized to β‐actin, and relative expression levels were calculated using the 2^−ΔΔCt method, with HET‐1A cells used as the reference. Primer sequences, synthesized by Sangon Biotech (Shanghai, China), are listed in Table S2. The methods for drug sensitivity prediction and molecular docking analysis of *GNGT1* are described in Methods S3.

### 2.7 Statistical Analysis

All statistical analyses were performed using R software (v4.3.2) and OmicsTools software (https://github.com/zihaoxingstudy1/). For continuous variables: *t*‐test or one‐way ANOVA for normal distribution; Wilcoxon rank‐sum test or Kruskal–Wallis test for non‐normal distribution. Chi‐square test or Fisher's exact test was used for categorical variables. Survival analysis was performed using the Kaplan–Meier method to plot survival curves, and the log‐rank test to compare survival differences between groups. Univariate Cox proportional hazards regression was used to assess prognostic correlation. For all multi‐comparison settings, Benjamini–Hochberg FDR adjustment was applied to control the false discovery rate. Statistical significance: *p < 0.05, **p < 0.01, ***p < 0.001, ****p < 0.0001.

## 3 Results

### 3.1 Single‐Cell Transcriptomic Landscape of the ESCC Tumor Microenvironment and Identification of Malignant Epithelial Cells

**FIGURE 1:**
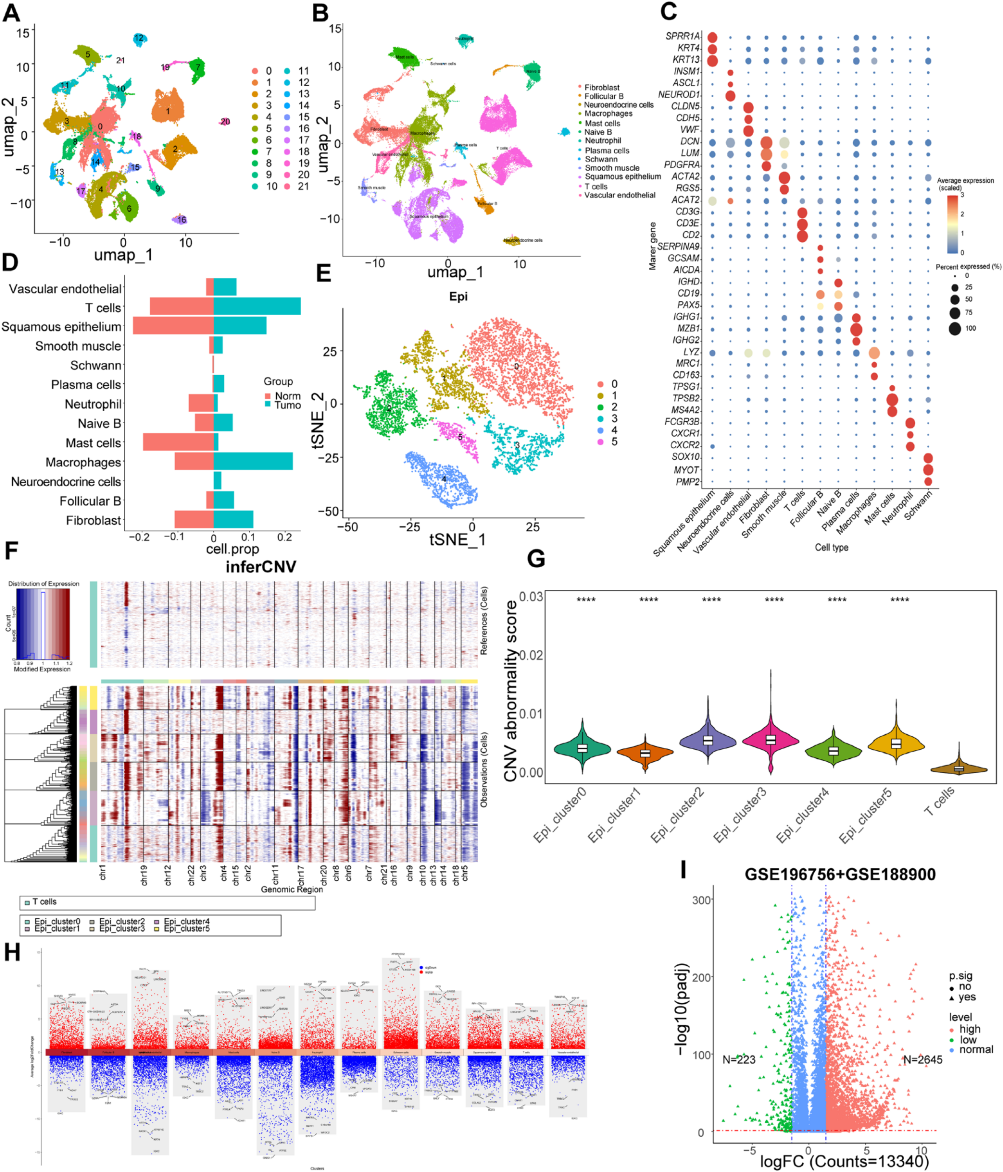
Single‐cell transcriptomic landscape of the ESCC tumor microenvironment and identification of malignant epithelial populations. (A) UMAP plot showing 21 cell subsets after integration of the GSE196756 and GSE188900 datasets. (B) UMAP plot showing annotated major cell types. (C) Bubble plot showing the expression levels and proportions of representative marker genes across major cell types. (D) Bar plot showing the cellular composition of tumor and normal tissues. (E) UMAP plot showing six epithelial subclusters after epithelial cell re‐clustering. (F) inferCNV heatmap showing inferred copy‐number variation profiles of epithelial subclusters across chromosomes. (G) Violin plot comparing CNV scores between epithelial subclusters and T cells. (H) Volcano plot showing differentially expressed genes across cell populations. (I) Volcano plot showing differentially expressed genes between malignant epithelial and normal tissue‐derived epithelial cells.

To characterize the cellular heterogeneity of the ESCC tumor microenvironment, we integrated two scRNA‐seq datasets (GSE196756 and GSE188900). After batch‐effect correction, 10 tumor and 4 adjacent normal tissues were analyzed. UMAP clustering identified 21 cell subsets (Figure 1A), annotated based on canonical marker genes, including fibroblasts, follicular B cells, neuroendocrine cells, macrophages, mast cells, naive B cells, neutrophils, plasma cells, Schwann cells, smooth muscle cells, squamous epithelial cells, T cells, and vascular endothelial cells (Figure 1B). The expression patterns of representative marker genes further confirmed these annotations (Figure 1C). Cellular composition differed markedly between tumor and normal tissues, with T cells, squamous epithelial cells, and macrophages predominant in the ESCC microenvironment (Figure 1D), suggesting their potential roles in tumor immunity and stromal remodeling.

To further characterize malignant epithelial populations, we extracted and re‐clustered epithelial cells into six subclusters (Figure 1E). Using T cells as references, inferCNV revealed widespread chromosomal CNV patterns (Figure 1F), and CNV scores were significantly higher in all epithelial subclusters than in T cells, confirming their malignant identity (Figure 1G). Differential expression analysis revealed extensive transcriptional heterogeneity (Figure 1H). Malignant epithelial cells showed 1645 upregulated and 223 downregulated genes versus normal epithelial cells (Figure 1I), providing the basis for subsequent biomarker identification.

### 3.2 Integrated Bulk and Single‐Cell Transcriptomic Analyses Identify Malignant Epithelial Cell‐Associated Biomarkers in ESCC

**FIGURE 2:**
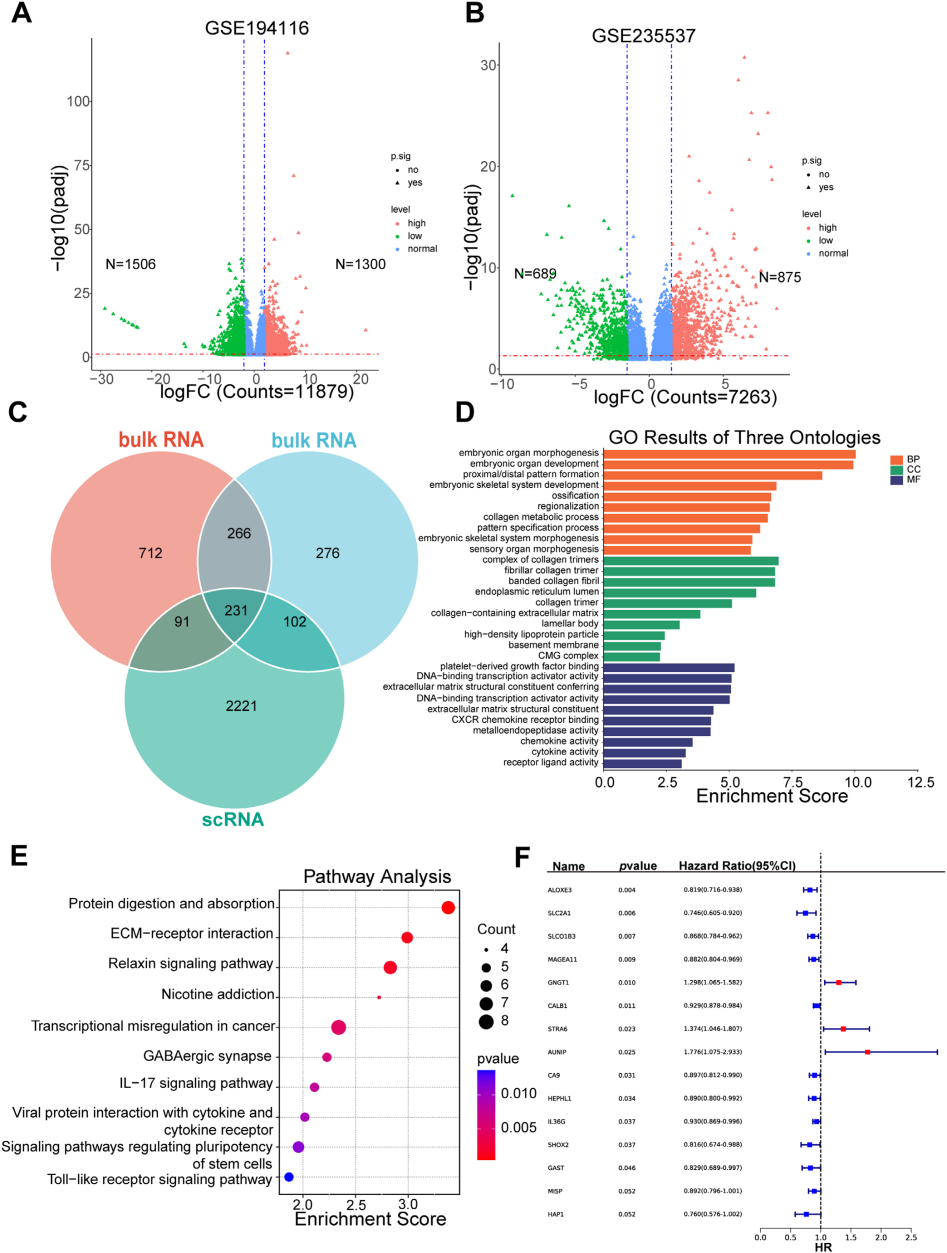
Identification of malignant epithelial cell‐associated candidate biomarkers in ESCC. (A, B) Volcano plot depicting DEGs in the GSE194116 and GSE235537 datasets. (C) Venn diagram illustrating the overlap between bulk RNA‐seq and scRNA‐seq datasets. (D) GO functional enrichment analysis of the 231 candidate genes. (E) KEGG pathway enrichment analysis of the 231 candidate genes. (F) Forest plot depicting univariate Cox regression analysis results of the 231 genes.

To identify malignant epithelial cell‐associated genes, we performed differential expression analysis in two independent bulk RNA‐seq datasets. GSE194116 yielded 1300 upregulated and 1506 downregulated genes (Figure 2A), while GSE235537 yielded 875 upregulated and 689 downregulated genes (Figure 2B). Intersecting the upregulated genes from both datasets with the 1645 malignant epithelial cell‐enriched genes from single‐cell analysis identified 231 overlapping genes, defined as malignant epithelial cell‐associated candidates (Figure 2C).

GO enrichment analysis showed that these candidates were enriched in terms related to embryonic organ morphogenesis and development, fibrillar collagen trimer, DNA‐binding transcription activator activity, chemokine activity, and cytokine activity (Figure 2D). KEGG analysis further revealed enrichment in ECM‐receptor interaction, protein digestion and absorption, IL‐17 signaling, and Toll‐like receptor signaling pathways (Figure 2E), suggesting involvement in extracellular matrix remodeling and immune‐related processes in ESCC.

To identify candidates with prognostic relevance, we performed univariate Cox regression analysis for the 231 genes in the GSE53625 cohort. Thirteen genes, *ALOXE3, SLC2A1, SLCO1B3, MAGEA11, GNGT1, CALB1, STRA6, AUNIP, CA9, HEPHL1, IL36G, SHOX2*, and *GAST*, were significantly associated with overall survival (Figure 2F) and retained for subsequent machine‐learning‐assisted prioritization.

### 3.3 Machine Learning‐Assisted Prioritization of Candidate Biomarkers

**FIGURE 3:**
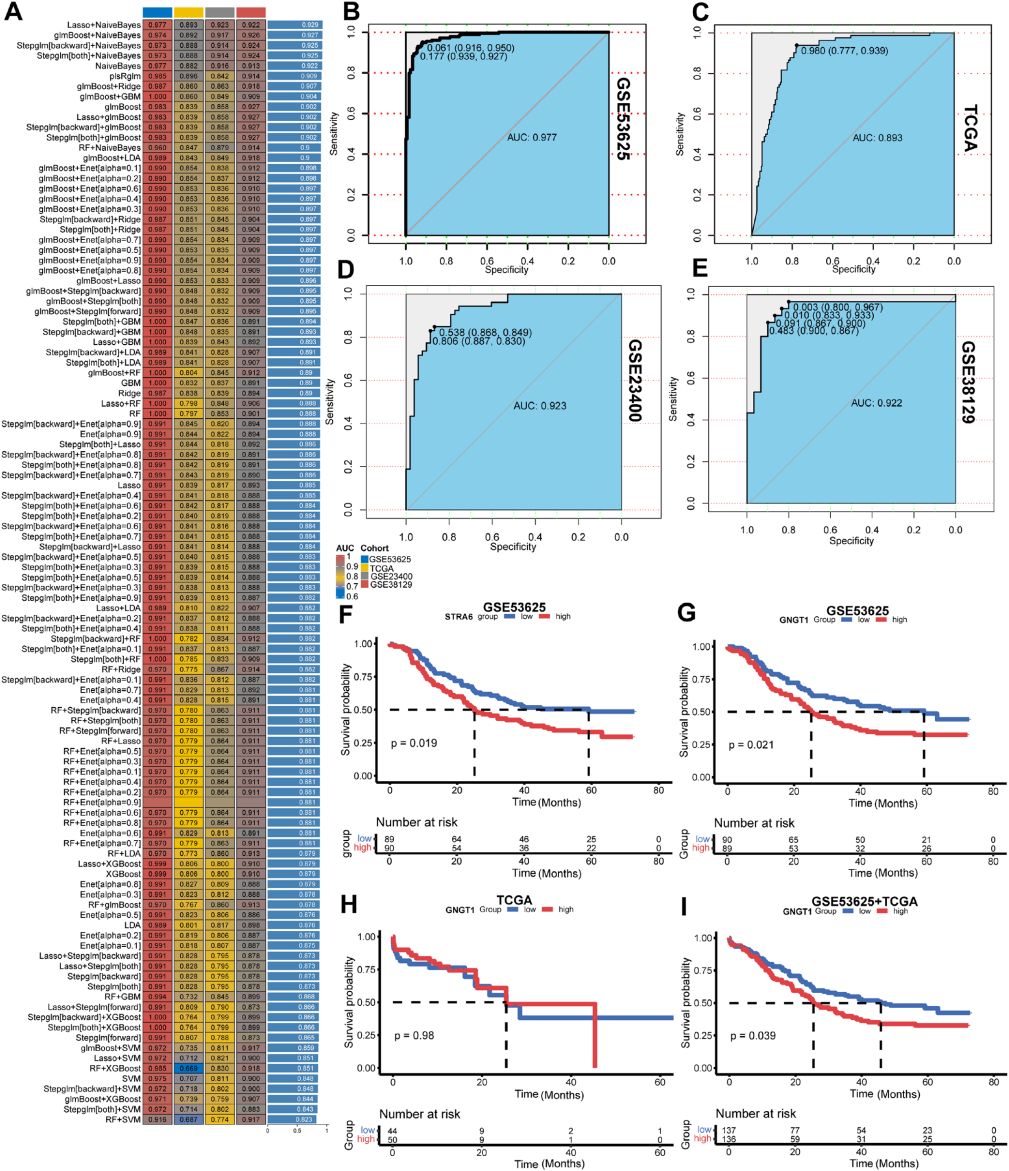
Machine Learning‐Assisted Prioritization of Candidate Biomarkers. (A) AUC values of 113 machine learning algorithm combinations for diagnostic model construction. (B–E) ROC curves of the optimal diagnostic model (LASSO + naive Bayes) in the training set GSE53625 (B), and validation sets TCGA (C), GSE23400 (D), and GSE38129 (E). (F, G) KM curves of *STRA6* and *GNGT1* in the GSE53625 cohort. (H) KM curves of *GNGT1* in the TCGA‐ESCC cohort. (I) KM curves of *GNGT1* in the combined TCGA and GSE53625 cohorts.

Based on the 13 prognosis‐associated genes, we applied a machine‐learning‐assisted screening strategy to further prioritize candidates with diagnostic relevance. Among the 113 algorithm combinations, LASSO plus naive Bayes showed the highest average discriminatory performance across training and validation cohorts (Figure 3A). The AUC was 0.977 in the GSE53625 training cohort and 0.893, 0.923, and 0.922 in the three independent validation cohorts (TCGA‐ESCC, GSE23400, and GSE38129), respectively (Figure 3B–E), supporting the diagnostic relevance of the genes retained by this model.

Eleven candidate genes were retained: *SLC2A1, SLCO1B3, MAGEA11, GNGT1, CALB1, STRA6, AUNIP, CA9, IL36G, SHOX2*, and *GAST*. Kaplan–Meier survival analysis in the GSE53625 cohort showed that only *GNGT1* and *STRA6* were significantly associated with overall survival among these genes (Figure 3F–G). As *STRA6* has been extensively studied in multiple cancers while *GNGT1* remains poorly characterized in ESCC, we selected *GNGT1* for subsequent analyses.

In the TCGA‐ESCC cohort, *GNGT1* expression alone was not significantly associated with overall survival, possibly due to the limited ESCC sample size (Figure 3H). We therefore combined TCGA‐ESCC and GSE53625, yielding 273 ESCC patients for survival analysis. The results of batch effect removal are shown in Figure S1. In this combined cohort, patients with high *GNGT1* expression had significantly poorer overall survival than those with low expression (Figure 3I). Multivariate Cox regression further supported this association (Figure S2). Together, these findings prioritized *GNGT1* as a candidate biomarker with diagnostic and prognostic relevance in ESCC.

### 3.4 Multi‐Cohort Validation of *GNGT1* Expression, Diagnostic Performance, and Clinical Relevance

**FIGURE 4:**
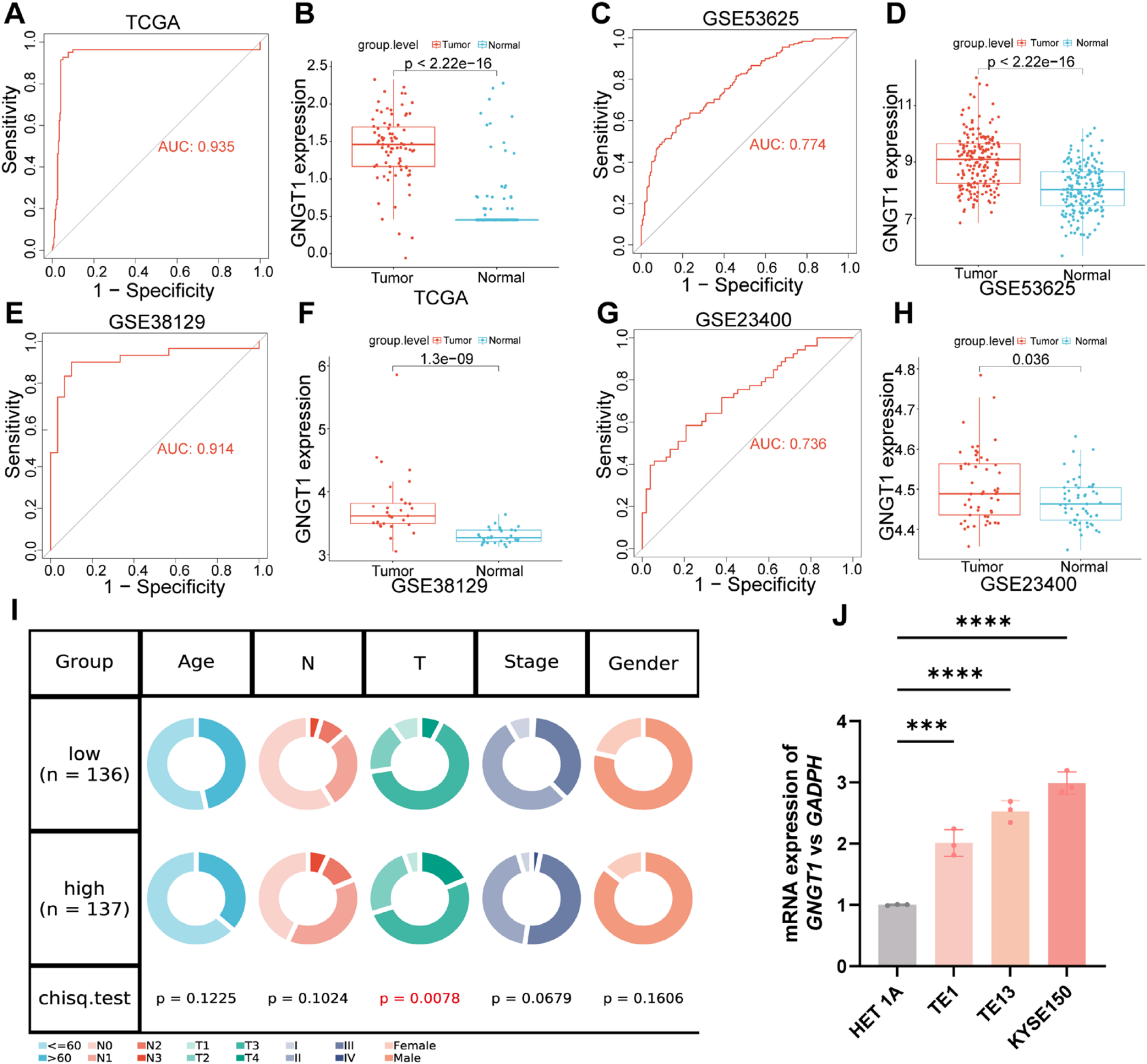
Clinicopathological features of *GNGT1* expression. (A–H) ROC curves and boxplots depicting *GNGT1* expression in tumor versus normal tissues. (A, B) TCGA cohort; (C, D) GSE53625 cohort; (E, F) GSE38129 cohort; (G, H) GSE23400 cohort. (I) Pie charts illustrating the distribution of clinicopathological features between *GNGT1* high‐ and low‐expression groups in the combined TCGA and GSE53625 cohort; (J) *GNGT1* mRNA expression in HET‐1A, TE1, TE13 and KYSE150 cells detected by qPCR. The results were analyzed using an unpaired *t*‐test. Data are presented as the mean ± SD, and significance is indicated. ***p < 0.001, ****p < 0.0001.

To further evaluate the expression pattern and clinical relevance of *GNGT1* in ESCC, we analyzed multiple independent cohorts. In the TCGA‐ESCC cohort, *GNGT1* showed favorable discriminatory performance between tumor and normal tissues (AUC = 0.935), with significantly higher expression in tumor samples (Figure 4A–B). Similar results were observed in the GSE53625, GSE38129, and GSE23400 cohorts, in which the AUC values of *GNGT1* were all above 0.7 and *GNGT1* expression was consistently higher in tumors than in normal tissues (Figure 4C–H). These findings support the diagnostic relevance of *GNGT1* in ESCC.

We next assessed the association between *GNGT1* expression and clinicopathological features in the combined TCGA‐ESCC and GSE53625 cohort. T stage distribution differed significantly between the *GNGT1*‐high and *GNGT1*‐low groups, with a higher proportion of T4‐stage patients in the *GNGT1*‐high group (Figure 4I). Proportions of patients with N1, N2, and N3 disease were also higher in the *GNGT1*‐high group, though the differences were not statistically significant.

Consistent with the cohort‐level findings, qPCR analysis showed that *GNGT1* expression was increased in ESCC cell lines compared with HET‐1A cells. Among the tested cell lines, KYSE150 showed the highest relative expression level (RQ = 2.85–3.19), followed by TE13 (RQ = 2.34–2.69) and TE1 (RQ = 1.81–2.24) (Figure 4J). Together, these results indicate that *GNGT1* is upregulated in ESCC and may be associated with more advanced local tumor status.

In addition, a pan‐cancer analysis of *GNGT1* was performed to evaluate its expression, prognostic value, genetic alterations, and immune correlations across multiple cancer types. The results are provided in Text S1 and Figure S3.

### 3.5 Immune Infiltration Analysis

**FIGURE 5:**
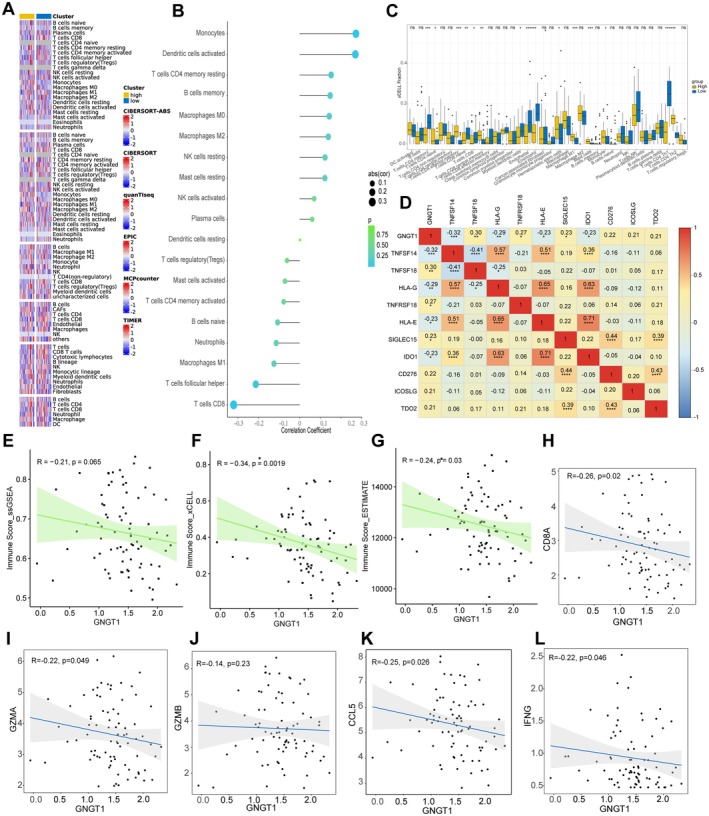
Immune infiltration analysis. (A) Comparison of immune cell infiltration between *GNGT1*‐high and *GNGT1*‐low groups using six immune infiltration algorithms. (B) Correlation between *GNGT1* expression and immune cell infiltration estimated by CIBERSORT. (C) Comparison of immune cell infiltration between *GNGT1*‐high and *GNGT1*‐low groups based on xCELL. (D) Heatmap showing correlations between *GNGT1* expression and immune checkpoint‐related molecules. (E–G) Correlations between *GNGT1* expression and immune scores calculated using ssGSEA, xCELL, and ESTIMATE. (H–L) Scatter plots showing correlations between *GNGT1* expression and CD8^+^ T cell marker genes.*p < 0.05, **p < 0.01, ***p < 0.001, ****p < 0.0001. NS, not significant (*p* > 0.05).

Pan‐cancer analysis suggested that *GNGT1* may be associated with tumor immune microenvironment regulation and immunotherapy response. To investigate this in ESCC, we stratified patients into *GNGT1*‐high and *GNGT1*‐low groups based on median *GNGT1* expression and analyzed immune cell infiltration using six algorithms. The distribution of immune cell subpopulations differed significantly between the two groups (Figure 5A).

CIBERSORT analysis revealed that *GNGT1* expression was significantly negatively correlated with CD8^+^ T cells and M1 macrophages, and positively correlated with monocytes and dendritic cells (Figure 5B). Consistently, all six algorithms, including xCELL, showed significantly lower CD8^+^ T cell infiltration in the *GNGT1*‐high group (Figure 5C; Figure S4). These findings indicate that high *GNGT1* expression is significantly associated with reduced CD8^+^ T cell infiltration.

Immune checkpoint molecule analysis showed that GNGT1 expression was significantly correlated with *TNFSF14, TNFSF18, HLA‐G, HLA‐E*, and *SIGLEC15* (Figure 5D). Immune scores calculated by ssGSEA, xCELL, and ESTIMATE were all significantly negatively correlated with *GNGT1* expression (Figure 5E–G). Furthermore, *GNGT1* expression was significantly negatively correlated with the CD8^+^ T cell marker genes *CD8A, GZMA, CCL5*, and *IFNG* (Figure 5H–L), supporting its association with an immunosuppressive microenvironment signature.

### 3.6 Exploratory Analysis of *GNGT1* Expression and Treatment‐Related Features Across Multiple Cohorts

**FIGURE 6:**
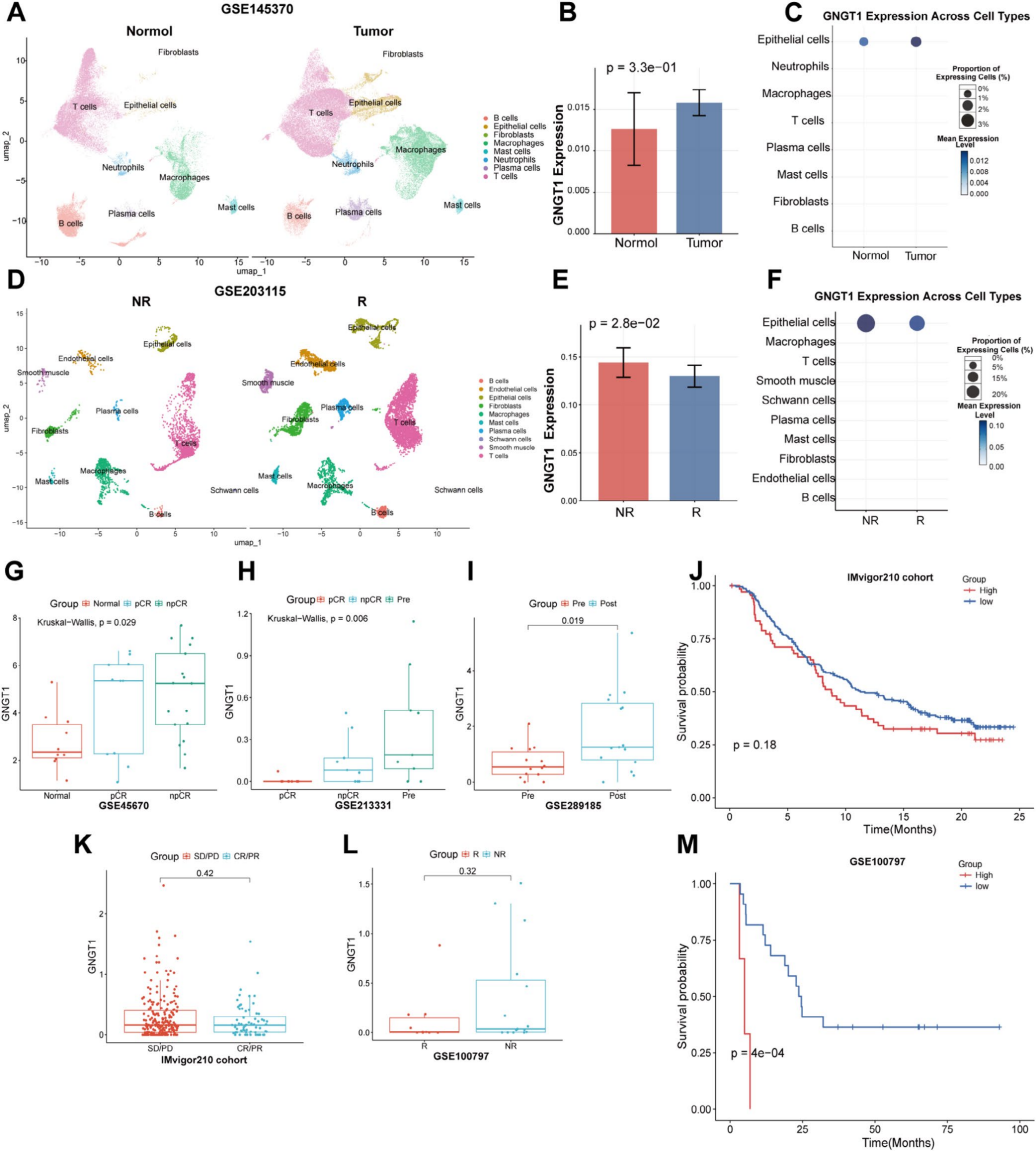
*GNGT1* expression and its association with treatment response across independent cohorts. (A) UMAP visualization of cell types in the GSE145370 dataset. (B) *GNGT1* expression in tumor versus normal tissues (GSE145370). (C) Cell‐type specific *GNGT1* expression in GSE145370. (D) UMAP of cell clusters in the GSE203115 cohort (ESCC post‐NACI). (E) *GNGT1* expression in NR versus R (GSE203115). (F) Cell‐type specific *GNGT1* expression in GSE203115. (G) *GNGT1* expression in the GSE45670 cohort (ESCC post‐CRT). (H) *GNGT1* expression in the GSE213331 cohort (rectal cancer post‐NAC). (I) *GNGT1* expression in the GSE289185 cohort (ESCC pre‐ versus post‐NACI). (J) KM survival curves for the IMvigor210 cohort. (K) *GNGT1* expression by response category in IMvigor210. (L) *GNGT1* expression by response in GSE100797 (advanced melanoma post‐immunotherapy). (M) KM survival curves for GSE100797.

Given the importance of neoadjuvant therapy response in esophageal cancer management, we systematically examined *GNGT1* expression across multiple independent neoadjuvant therapy cohorts. In the independent single‐cell dataset GSE145370, UMAP clustering identified eight major cell subpopulations (Figure 6A). *GNGT1* expression was significantly higher in tumor than in normal tissues (Figure 6B) and was predominantly restricted to epithelial cells, with significantly higher expression in tumor‐derived epithelial cells (Figure 6C). In the ESCC neoadjuvant immunochemotherapy (NACI) cohort GSE203115, UMAP clustering identified 10 cell subpopulations (Figure 6D). *GNGT1* expression showed a trend toward higher levels in the single non‐responder (NR) sample than in the two responder (R) samples; however, given the extremely limited sample size, this observation is strictly preliminary and no statistical inference can be drawn (Figure 6E). Bubble plots confirmed predominant expression in epithelial cells, with higher levels in the NR group (Figure 6F).

In the ESCC neoadjuvant chemoradiotherapy (CRT) cohort GSE45670, *GNGT1* expression was higher in both non‐pathological complete response (npCR) and pathological complete response (pCR) groups compared to normal tissues. There was an increasing trend in the npCR group relative to the pCR group (Figure 6G). In the rectal cancer neoadjuvant chemotherapy (NAC) cohort GSE213331, the highest *GNGT1* expression was observed in the pre‐treatment group. It was followed by the npCR group, with the lowest expression in the pCR group (Figure 6H). In the ESCC NACI cohort GSE289185, *GNGT1* expression was significantly higher in the post‐treatment group than in the pre‐treatment group (Figure 6I). We further analyzed advanced cancer immunotherapy cohorts. In the bladder cancer immunotherapy cohort IMvigor210, *GNGT1* overall expression was relatively low. However, survival analysis revealed a trend toward worse prognosis in the high‐expression group (Figure 6J). *GNGT1* expression was significantly higher in the stable disease/progressive disease (SD/PD) group than in the complete response/partial response (CR/PR) group (Figure 6K). In the advanced melanoma immunotherapy cohort GSE100797, no significant difference in *GNGT1* expression was observed between NR and R groups (Figure 6L). However, survival analysis indicated that patients with high *GNGT1* expression had poorer overall survival (Figure 6M). Collectively, these observations suggest a potential association between *GNGT1* expression and treatment resistance; however, the findings should be interpreted cautiously given the limited sample size and cross‐cohort heterogeneity.

### 3.7 Cell–Cell Communication Analysis of *GNGT1* ‐High Epithelial Cells

To explore the potential role of *GNGT1*‐high epithelial cells in intercellular communication within the ESCC tumor microenvironment, CellChat analysis was performed using two scRNA‐seq datasets: the primary ESCC cohort GSE145370 and the neoadjuvant immunochemotherapy cohort GSE203115.

**FIGURE 7:**
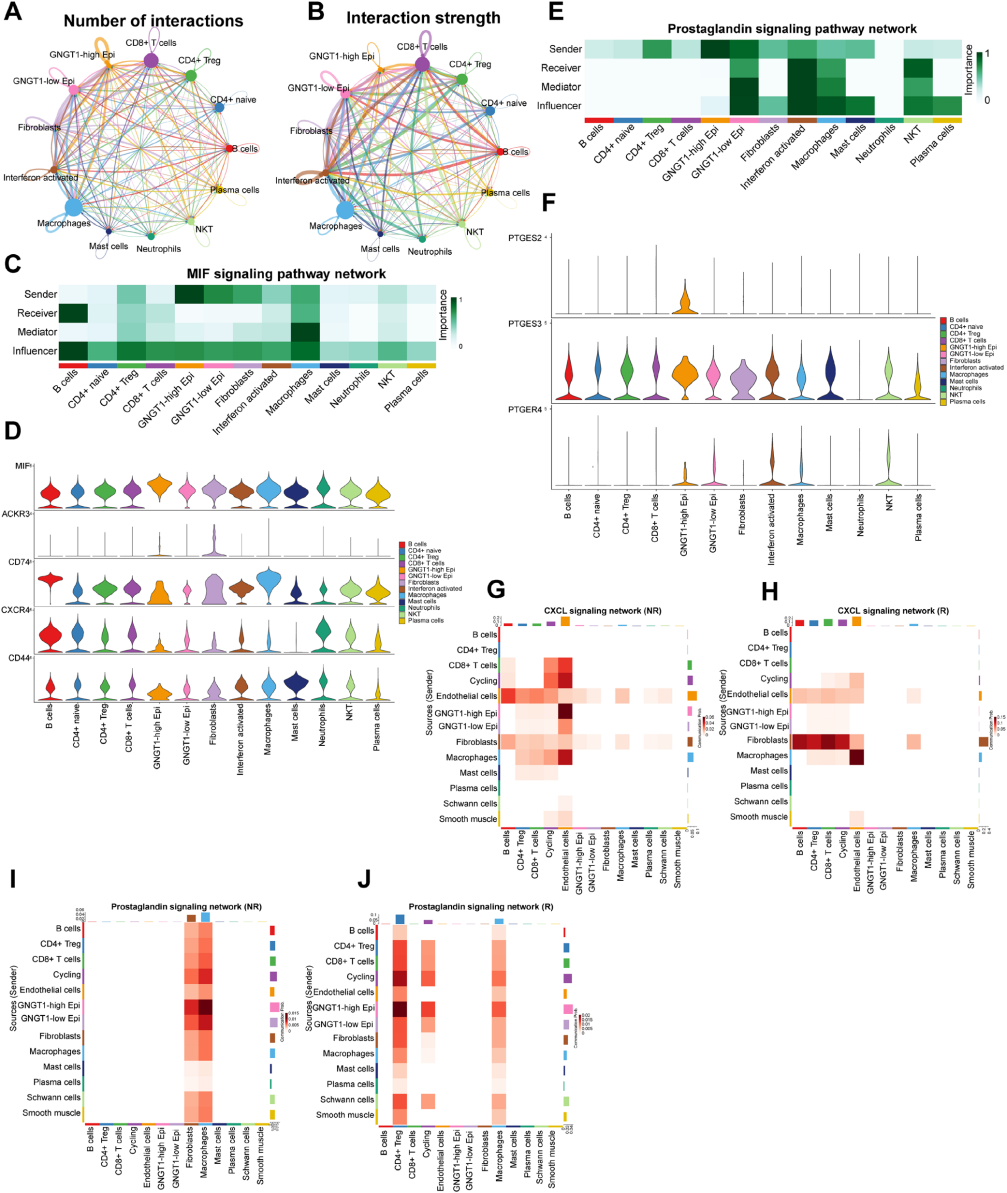
Cell–cell communication patterns associated with *GNGT1*‐high epithelial cells. (A, B) Circle plots showing the number and strength of inferred interactions among cell populations in the GSE145370 primary ESCC cohort. (C) Heatmap showing the inferred cell‐type‐specific communication network of the MIF signaling pathway. (D) Violin plots showing the expression of key ligand–receptor pairs involved in MIF signaling. (E) Heatmap showing the inferred cell‐type‐specific communication network of the prostaglandin signaling pathway. (F) Violin plots showing the expression of key ligand–receptor pairs involved in prostaglandin signaling. (G, H) Heatmaps showing CXCL signaling networks in non‐responder and responder samples from the GSE203115 cohort. (I, J) Heatmaps showing prostaglandin signaling networks in non‐responder and responder samples from the GSE203115 cohort.

In the primary ESCC cohort, CellChat inferred extensive intercellular communication among epithelial, immune, and stromal populations (Figure 7A,B). *GNGT1*‐high epithelial cells showed prominent interaction numbers and interaction strength with CD8^+^ T cells, CD4^+^ Tregs, macrophages, fibroblasts, and other immune or stromal cells, whereas *GNGT1*‐low epithelial cells showed relatively weaker communication patterns.

Pathway‐level analysis identified MIF signaling as a major outgoing pathway associated with *GNGT1*‐high epithelial cells. B cells and macrophages were predicted to be the main recipient cell populations (Figure 7C). Expression analysis of ligand–receptor pairs showed higher expression of MIF‐related components in *GNGT1*‐high epithelial cells, including *MIF, ACKR3, CD74, CXCR4*, and *CD44* (Figure 7D). These findings suggest that *GNGT1*‐high epithelial cells may participate in MIF‐mediated epithelial–immune communication.

A similar pattern was observed for prostaglandin signaling. *GNGT1*‐high epithelial cells were predicted to act as major signal senders, with interferon‐activated cells and macrophages identified as potential recipient populations (Figure 7E). Key prostaglandin‐related molecules, including *PTGES2, PTGES3*, and *PTGER4*, showed higher expression in *GNGT1*‐high epithelial cells than in *GNGT1*‐low epithelial cells (Figure 7F). These results indicate that *GNGT1*‐high epithelial cells are associated with enhanced MIF‐ and prostaglandin‐related communication.

We further examined cell–cell communication patterns in the NACI‐treated ESCC cohort. In this dataset, CXCL and prostaglandin signaling were among the prominent pathways associated with *GNGT1*‐high epithelial cells. For CXCL signaling, *GNGT1*‐high epithelial cells in the non‐responder group were predicted to mainly communicate with endothelial cells (Figure 7G,H). In the responder group, macrophages appeared as major signal senders targeting endothelial cells, with broader crosstalk involving fibroblasts, B cells, CD8^+^ T cells, and CD4^+^ Tregs. For prostaglandin signaling, *GNGT1*‐high epithelial cells were predicted to communicate mainly with macrophages in the non‐responder group, whereas CD4^+^ Tregs were the main predicted recipient cells in the responder group (Figure 7I,J).

Together, these single‐cell communication analyses suggest that *GNGT1*‐high epithelial cells were predicted to have extensive ligand‐receptor interactions involving MIF‐, prostaglandin‐, and CXCL‐related pathways with immune and stromal cells.

### 3.8 Pathway Enrichment Analysis Reveals Key Biological Processes Associated With *GNGT1* ‐High Epithelial Cells

**FIGURE 8:**
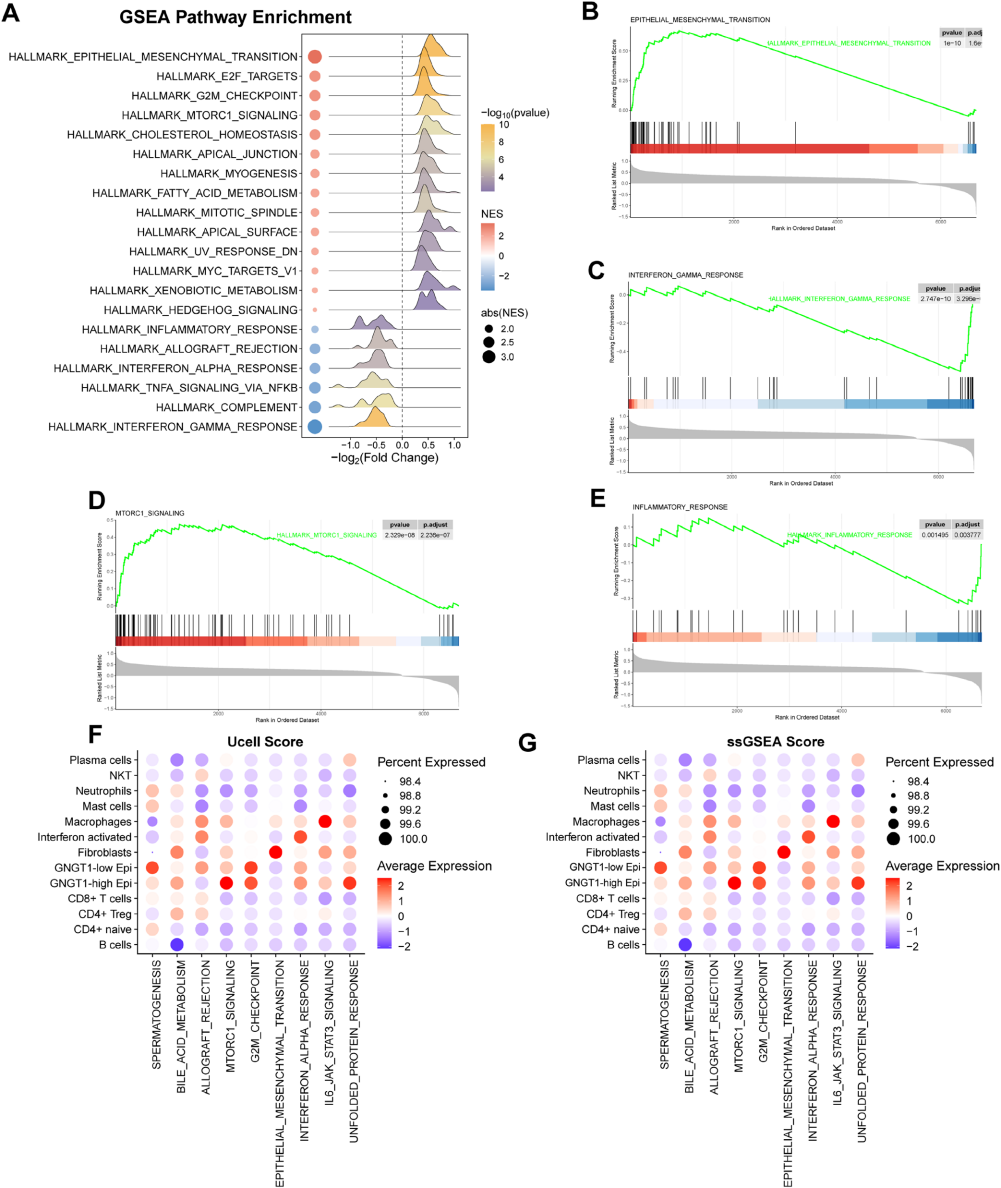
Pathway enrichment analysis of *GNGT1* in ESCC. (A) Hallmark pathway enrichment analysis based on *GNGT1* expression. (B–E) GSEA enrichment plots for epithelial‐mesenchymal transition, interferon gamma response, mTORC1 signaling, and inflammatory response. (F) Single‐cell pathway activity analysis based on UCell. (G) Single‐cell pathway activity analysis based on ssGSEA.

To investigate biological programs associated with *GNGT1* expression in ESCC, we performed GSEA stratified by *GNGT1* expression and assessed single‐cell pathway activity using UCell and ssGSEA. GSEA revealed enrichment of several hallmark pathways in the *GNGT1*‐high group. Epithelial–mesenchymal transition showed the strongest enrichment (NES = 3.26, *p* < 0.0001), followed by proliferation‐related pathways, including E2F targets, G2M checkpoint, and mTORC1 signaling. In contrast, immune‐related pathways, including interferon‐gamma response, inflammatory response, complement, and TNF‐α signaling via NF‐κB, were enriched in the *GNGT1*‐low group (Figure 8A–E). These findings are consistent with the immune infiltration analyses and suggest that high *GNGT1* expression is associated with a less immune‐active tumor microenvironment.

To further assess the cell‐type specificity of these pathways, UCell and ssGSEA analyses were performed in the GSE145370 single‐cell dataset. Both approaches showed higher activity of tumor‐associated programs, including mTORC1 signaling, G2M checkpoint, and oxidative stress‐related pathways, in *GNGT1*‐high epithelial cells (Figure 8F–G). These findings suggest that *GNGT1*‐high epithelial cells are associated with enhanced malignant epithelial programs and reduced immune‐related pathway activity in ESCC. Additionally, we performed *in silico* drug sensitivity prediction and molecular docking analyses to preliminarily explore the pharmacological relevance of *GNGT1*. Full results are provided in Text S2 and Figure S5.

## 4 Discussion

ESCC is an aggressive malignancy with poor prognosis, and its progression is closely linked to microenvironment remodeling and immune escape. Single‐cell transcriptomics enables the dissection of intratumoral heterogeneity and the identification of cell‐state‐specific molecular features. In this study, we integrated single‐cell and bulk transcriptomic datasets to identify malignant epithelial cell‐associated biomarkers in ESCC. Through cross‐cohort differential expression analysis, prognostic evaluation, and machine‐learning‐assisted prioritization, *GNGT1* was selected for further investigation. Subsequent analyses showed that high *GNGT1* expression was associated with tumor epithelial enrichment, poorer survival, reduced CD8^+^ T cell infiltration, altered epithelial–immune communication, and treatment‐related features. These findings suggest that *GNGT1* marks an immunosuppressive malignant epithelial cell state in ESCC.

GNGT1 is a γ subunit of heterotrimeric G proteins and participates in G protein‐coupled receptor‐mediated signal transduction (Guo et al. 2026). Under physiological conditions, *GNGT1* expression is predominantly restricted to retinal photoreceptors, where it encodes the γ subunit of the rod photoreceptor G protein transducin (Chen et al. 2007). In most non‐ocular tissues, including normal esophageal epithelium, *GNGT1* expression is barely detectable or entirely absent, consistent with its highly specialized physiological role. Therefore, the substantial upregulation of *GNGT1* observed in ESCC tumor tissues likely represents an ectopic pathological event. This aberrant activation may be associated with tumor development and progression (O'Hayre et al. 2013). However, its direct mechanistic links remain to be experimentally validated. Previous studies have only reported aberrant *GNGT1* expression in a limited number of cancer types. For instance, Fan et al. identified *GNGT1* as a poor prognostic marker in lung adenocarcinoma (Fan et al. 2025). Huang et al. used bioinformatic analyses and suggested that *GNGT1* may serve as a potential prognostic and immunotherapeutic marker in gastric cancer (Huang et al. 2025). To date, the expression pattern, clinical significance, and biological function of *GNGT1* in ESCC remain largely uncharacterized.

In this study, *GNGT1* showed favorable discriminatory ability for distinguishing ESCC tumor tissues from normal tissues. Survival analysis further showed that high *GNGT1* expression was associated with shorter overall survival in the combined TCGA‐ESCC and GSE53625 cohort (*n* = 273). In addition, clinicopathological analysis indicated that the *GNGT1*‐high group contained a higher proportion of T4‐stage patients, suggesting a possible association between *GNGT1* expression and more advanced local tumor status. Consistent with the public transcriptomic data, qPCR analysis confirmed increased *GNGT1* expression in TE1, TE13, and KYSE150 ESCC cell lines compared with HET‐1A cells. Collectively, these findings support *GNGT1* as a malignant epithelial cell‐associated biomarker with diagnostic and prognostic relevance in ESCC.

Multidimensional immune infiltration analyses revealed a marked reduction of CD8^+^ T cells and a significant negative correlation with immune scores in the *GNGT1*‐high group. GSEA showed that interferon response, inflammatory response, and complement pathways were enriched in the *GNGT1*‐low group, supporting an association between high *GNGT1* expression and an immunosuppressive microenvironment. Previous studies have reported that G protein signaling components, such as G protein γ subunit 12 (GNG12), play a role in cancer immune evasion, proliferation, angiogenesis, and immunotherapy resistance (Alausa et al. 2022), providing a theoretical basis for our findings. In addition, *GNGT1* expression was significantly negatively correlated with MSI and TMB, suggesting that *GNGT1*‐high tumors may display a “cold tumor” phenotype with reduced immunotherapy responsiveness, which may inform patient stratification in ESCC immunotherapy.

*GNGT1*‐high malignant epithelial cells act as a central signaling hub in the ESCC tumor microenvironment, with intensive crosstalk with CD8^+^ T cells, macrophages, and fibroblasts. Our computational analyses predicted that *GNGT1*‐high epithelial cells were enriched in ligand‐receptor interactions involving the MIF and prostaglandin pathways. MIF is a key pro‐inflammatory cytokine that inhibits CD8^+^ T cell activation and proliferation and drives macrophage polarization toward the M2 phenotype by binding to the CD74/CD44 receptor complex, thus mediating tumor immune escape (O'Reilly et al. 2016). Our results showed that *GNGT1*‐high epithelial cells highly expressed the MIF ligand and its receptors ACKR3/CD74, with macrophages and B cells as the main signal‐receiving cells. Meanwhile, key molecules of the prostaglandin pathway, including PTGES2/PTGES3 (PGE2 synthases) and the receptor PTGER4, were upregulated in *GNGT1*‐high epithelial cells. PGE2 is known to restrict the proliferation and effector differentiation of tumor‐infiltrating CD8^+^ T cells via EP2 and EP4 signaling, promoting cancer immune evasion (Lacher et al. 2024).

We further explored cell–cell communication patterns in the context of neoadjuvant immunochemotherapy. In the non‐responder group, *GNGT1*‐high epithelial cells were predicted to communicate primarily with endothelial cells through CXCL signaling, suggesting a possible association with vascular remodeling and immune cell trafficking under treatment‐resistant conditions. In the responder group, macrophages appeared to be the major senders of CXCL signals to endothelial cells, accompanied by broader crosstalk among fibroblasts, B cells, CD8^+^ T cells, and CD4^+^ Tregs. These observations are consistent with previous reports that CXCL chemokines are involved in tumor immune regulation and therapeutic response (Sadhukhan et al. 2024). Across multiple therapy‐related cohorts, exploratory analyses suggested a potential association between *GNGT1* expression and treatment‐related outcomes. However, these findings should be interpreted cautiously because of small sample sizes, heterogeneous treatment settings, and the limited number of non‐responder samples in the single‐cell NACI cohort.

Pathway enrichment analyses showed that *GNGT1*‐high epithelial cells were associated with EMT, mTORC1 signaling, and oxidative stress‐related programs. These pathways may reflect a malignant epithelial cell state characterized by enhanced proliferative, invasive, and metabolic features. EMT is closely linked to tumor invasion, metastasis, and immune suppression (Zhang and Stuelten 2024), while aberrant mTORC1 signaling has been implicated in ESCC progression, poor prognosis, and therapeutic resistance (Kim et al. 2017). In addition, drug sensitivity prediction suggested that *GNGT1*‐high samples may show lower predicted IC50 values for several targeted agents. Molecular docking analysis further suggested potential interactions between *GNGT1* and selected compounds, including ibrutinib and taselisib. Nevertheless, these results remain computational and hypothesis‐generating. Experimental validation is required to determine whether *GNGT1* directly affects drug response or can be therapeutically targeted in ESCC.

Several limitations should be acknowledged. First, this study was primarily based on retrospective public datasets, and prospective clinical validation is lacking. Second, although multi‐cohort transcriptomic analyses supported the clinical and immune relevance of *GNGT1*, its causal role in ESCC progression and immune microenvironment regulation remains to be determined through in vitro and in vivo experiments. Third, the drug sensitivity and molecular docking analyses were based on computational predictions; pharmacological validation is required to assess whether *GNGT1* expression correlates with drug response. Fourth, the neoadjuvant immunochemotherapy single‐cell cohort had a limited sample size, with only one non‐responder sample, which restricts the robustness of treatment‐response‐related conclusions. Finally, some immunotherapy‐related analyses were performed in bladder cancer and melanoma cohorts, and their findings cannot be directly extrapolated to ESCC.

Future studies should validate *GNGT1* expression and its associations with clinicopathological features, immune infiltration, and prognosis in larger ESCC clinical cohorts. Functional experiments are also needed to determine whether *GNGT1* is functionally involved in malignant epithelial programs, MIF/prostaglandin‐related communication, or CD8^+^ T cell‐associated immune suppression. In addition, ESCC‐specific treatment cohorts and experimental drug‐response assays will be important for evaluating the potential clinical utility of *GNGT1* in therapeutic stratification.

## 5 Conclusion

This study identifies *GNGT1* as a malignant epithelial cell‐associated candidate biomarker in ESCC through integrated single‐cell and bulk transcriptomic analyses. *GNGT1* was upregulated in ESCC tissues and cell lines and was associated with tumor–normal discriminatory ability, poorer overall survival, reduced CD8^+^ T cell infiltration, and altered MIF/prostaglandin‐related epithelial–immune communication. These findings suggest that *GNGT1* expression is associated with an immune‐suppressed malignant epithelial state in ESCC. Further experimental and clinical validation is required to clarify the functional role and translational relevance of *GNGT1*.

## Author Contributions

**Yintao Chang:** formal analysis. **Lei Xue:** conceptualization, supervision, project administration. **Qian Yuan:** formal analysis, writing – original draft, writing – review and editing. **Yuxiang Jin:** conceptualization, supervision, project administration. **Cheng Wang:** visualization, writing – review and editing. **Qiang Lyu:** formal analysis.

## Funding

The authors have nothing to report.

## Ethics Statement

The authors have nothing to report.

## Consent

All authors have read the manuscript and agreed to publish.

## Conflicts of Interest

The authors declare no conflicts of interest.

## Supporting information


**Methods S1.** Detailed data preprocessing, normalization, and batch‑effect correction.
**Methods S2**. Pan‐cancer analysis.
**Methods S3**. Drug sensitivity prediction and molecular docking analysis.
**Figure S1:** PCA plots before and after ComBat batch correction.
**Figure S2:** Multivariate Cox analysis of *GNGT1* expression.
**Figure S3:** Pan‐cancer analysis of *GNGT1*.
**Figure S4:** Six immune infiltration analyses.
**Figure S5:** Drug sensitivity prediction and molecular docking analysis associated with *GNGT1* expression.
**Table S1:** The information of public datasets in the study.
**Table S2:** Primer Sequences for qPCR.

## Data Availability

All data used in this work can be obtained from the Gene Expression Omnibus (GEO) datasets (https://www.ncbi.nlm.nih.gov/geo/) and the Cancer Genome Atlas (TCGA) datasets (https://xenabrowser.net/). Code and processed data: Available from the corresponding author upon reasonable request.
