# Supplementary material for: Single‐Cell and Bulk Transcriptomics Identify GNGT1‐High Malignant Epithelial Cells Associated With Immune Suppression in Esophageal Squamous Cell Carcinoma
Source: Chem Biol Drug Des. 2026 Aug 12;108(2):e70381. doi: 10.1111/cbdd.70381 (PMC13469733; doi:10.1111/cbdd.70381)
Supplement: Supplementary file 1 [file CBDD-108-e70381-s001.docx]

**Supplementary Methods S1. Detailed Data Preprocessing, Normalization, and Batch‑Effect Correction**

Data sources and quality control

All transcriptomic and microarray datasets used in this study were obtained from public repositories (Gene Expression Omnibus and The Cancer Genome Atlas) and were re‑analyzed by us; no newly generated transcriptomic data were used in the discovery or validation phases (newly generated qPCR data are described separately in the main Methods Section 2.6). Four independent ESCC scRNA‑seq datasets and ten bulk transcriptome or microarray datasets were included, covering a total of 1,318 samples. Detailed dataset information, including GEO/TCGA accession IDs, platform types, sample sizes, and specific usage (discovery, training, or validation), are summarized in Table S1.

For raw RNA‑seq count data, low‑expression genes were filtered using the following criteria: genes with counts per million (CPM) < 1 in more than 50% of samples were excluded. For microarray data, probes with low detection flags were removed. Sample‑level quality control (QC) included assessment of RNA integrity numbers (RINs) where available, and visual inspection of density plots and principal component analysis (PCA) for outlier detection.

Normalization and differential expression analysis

DESeq2 (v1.40.2, median‑ratio normalization) and limma (v3.58.1) were used for differential expression analysis according to experimental design [1]. The limma pipeline adopted TMM normalization followed by voom transformation to fit the distribution of RNA‑seq data. Microarray data were normalized using the RMA method. All adjusted P-values reported for differential expression were Benjamini–Hochberg false discovery rate (FDR) adjusted. Differentially expressed genes (DEGs) were identified with thresholds of |log₂FC| ≥ 1 and FDR < 0.05. Transcriptomic quantitative expression values were transformed as log₂(TPM+1) where applicable [2].

Batch‑effect correction

Batch‑effect correction with ComBat was applied exclusively for the purpose of merging the TCGA‑ESCC and GSE53625 cohorts for combined Kaplan–Meier survival analysis, to minimize technical batch effects between these two distinct RNA‑seq cohorts before pooling. The ComBat algorithm (from the sva package v3.48.0) was run with default empirical Bayes parameters (mean‑only adjustment for small batch sizes when applicable)[3]. The effectiveness of batch removal was verified by PCA plots before and after correction (Figure S5A‑B).

For all other analyses, including differential expression (Section 3.2), machine‑learning model training and diagnostic ROC validation (Sections 3.3–3.4), and immune infiltration (Section 3.5), analyses were performed within individual datasets or using within‑dataset normalization, without any cross‑dataset ComBat adjustment. This design ensures that the core findings are not confounded by batch‑correction procedures.

**Supplementary Methods S2. Pan-Cancer Analysis**

Normalized RNA-seq data, clinical survival data, tumor mutation burden (TMB), and microsatellite instability (MSI) data for 33 cancer types were downloaded from the UCSC Xena platform. Analyses and visualizations were performed using the R package TCGAplot and OmicsTools software (https://github.com/zihaoxingstudy1/), including: pan-cancer expression differences of *GNGT1* between tumor and paired normal tissues; correlation between *GNGT1* expression and overall survival of each cancer type by univariate Cox regression; correlations of *GNGT1* expression with 22 immune cell infiltration abundances, TMB, and MSI[4]. *GNGT1* pan-cancer genetic alteration data were obtained from the cBioPortal database (https://www.cbioportal.org/), and alteration frequency and type were statistically analyzed and visualized.

**Supplementary Methods S3. Drug sensitivity prediction and molecular docking analysis**

Drug sensitivity was estimated using oncoPredict (v1.2) based on drug response data from the CTRP and GDSC2 databases[5]. The predicted half-maximal inhibitory concentration (IC50) values of small-molecule compounds were calculated for ESCC samples, with higher IC50 values indicating lower predicted drug sensitivity. Spearman correlation analysis was then performed to evaluate the association between *GNGT1* expression and predicted drug IC50 values. Candidate drugs were selected using thresholds of |correlation coefficient| > 0.2 and P < 0.05.

Molecular docking was further performed as an exploratory analysis for selected candidate compounds. The three-dimensional structure of *GNGT1* was obtained from the RCSB Protein Data Bank, and ligand structures in SDF format were obtained from PubChem. Docking simulations were conducted using AutoDock Vina (v1.2.3), with the grid box covering the predicted binding pocket of *GNGT1*[6]. Binding affinity was evaluated using binding free energy in kcal/mol, and docking conformations were visualized using PyMOL (v2.5.0).

**Reference**

1.	Johnson W E, Li C, Rabinovic A.Adjusting batch effects in microarray expression data using empirical Bayes methods. Biostatistics 2007, 8(1):118-127.https://doi.org/10.1093/biostatistics/kxj037

2.	Liu S, Wang Z, Zhu R et al.Three Differential Expression Analysis Methods for RNA Sequencing: limma, EdgeR, DESeq2. J Vis Exp 2021(175).https://doi.org/10.3791/62528

3.	Zhao Y, Li M C, Konaté M M et al.TPM, FPKM, or Normalized Counts? A Comparative Study of Quantification Measures for the Analysis of RNA-seq Data from the NCI Patient-Derived Models Repository. J Transl Med 2021, 19(1):269.https://doi.org/10.1186/s12967-021-02936-w

4.	Yang P, Yin J, Zhang G et al.Comprehensive pan-cancer analysis identified SLC16A3 as a potential prognostic and diagnostic biomarker. Cancer Cell Int 2025, 25(1):168.<https://doi.org/10.1186/s12935-025-03791-1>

5.	Pan S, Hu W, Xie P et al.Single-cell and multi-omics integrative modeling identifies mitochondrial gene HSPE1 as a therapeutic target in osteosarcoma. J Transl Med 2026, 24(1):142.<https://doi.org/10.1186/s12967-025-07633-6>

6.	Tian F, He X, Wang S et al.Integrating single-cell sequencing and machine learning to uncover the role of mitophagy in subtyping and prognosis of esophageal cancer. Apoptosis 2025, 30(3-4):1021-1041.<https://doi.org/10.1007/s10495-024-02061-1>

**Figure S1 PCA plots before and after ComBat batch correction.** (A) PCA plot of the merged TCGA-ESCC and GSE53625 cohorts before batch correction, showing clear separation between datasets. (B) PCA plot after ComBat correction, demonstrating effective batch effect reduction.

 **Figure S2 Multivariate Cox analysis of *GNGT1* expression.**

**Text S1. Pan-cancer Analysis of *GNGT1***

To comprehensively evaluate the expression characteristics and biological significance of *GNGT1* across different cancers, we conducted systematic pan-cancer analyses. *GNGT1* was significantly upregulated in 17 cancer types compared to normal tissues (Figure S3A). These included bladder urothelial carcinoma (BLCA), breast invasive carcinoma (BRCA), esophageal carcinoma (ESCA), and head and neck squamous cell carcinoma (HNSC). This finding suggests that *GNGT1* exhibits specific upregulation across multiple tumor types.

Regarding prognostic value, pan-cancer survival analysis was performed. Results showed that *GNGT1* expression was significantly associated with overall survival in 5 cancer types (Figure S3B). These included adrenocortical carcinoma (ACC), liver hepatocellular carcinoma (LIHC), lung adenocarcinoma (LUAD), pheochromocytoma and paraganglioma (PCPG), and uterine corpus endometrial carcinoma (UCEC). This indicates *GNGT1*’s potential as a cross-cancer prognostic biomarker.

Genetic alteration features were analyzed using the cBioPortal platform. Results revealed that *GNGT1* alterations were dominated by gene amplification. Across six cancer types, the alteration frequency of *GNGT1* exceeded 5% (Figure S3C). ESCA had the highest frequency at 13.68%, which was predominantly driven by amplification. Immune correlation analysis further demonstrated that the association between *GNGT1* expression and immune cell infiltration varied by cancer type. In ESCA, *GNGT1* expression was positively correlated with macrophages, dendritic cells, and CD4+ naive T cells. It was negatively correlated with regulatory T cells (Tregs), naive B cells, and CD8+ T cells (Figure S3D).

Moreover, across multiple cancer types including ESCA, *GNGT1* expression was significantly negatively correlated with both MSI and TMB (Figure S3E–F). These findings suggest a potential association between *GNGT1* expression and tumor immunogenicity, although its relevance to immunotherapy response requires further validation.

**Figure S3 Pan-cancer analysis of *GNGT1*.** (A) Boxplot depicting *GNGT1* expression differences between tumor and normal tissues across multiple cancer types. (B) Forest plot displaying the association between *GNGT1* expression and overall survival across various cancer types. (C) Bar plot illustrating the frequency and types of *GNGT1* genetic alterations across various cancer types. (D) Heatmap depicting correlations between *GNGT1* expression and 22 immune cell types across different cancers. (E-F) Radar plot depicting the correlation between *GNGT1* expression and MSI and TMB.

**Figure S4 Six immune infiltration analyses.**

**Text S2. Drug sensitivity prediction and molecular docking analysis associated with *GNGT1* expression**

To explore the clinical translational potential of high *GNGT1* expression, drug sensitivity prediction was performed using CTRP and GDSC2 data. Candidate compounds were screened according to the correlation between *GNGT1* expression and predicted IC50 values, with thresholds of |correlation coefficient| > 0.2 and *P* < 0.05.

*GNGT1* expression was negatively correlated with the predicted IC50 values of multiple compounds, suggesting that *GNGT1*-high samples may have higher predicted sensitivity to these agents (Figure S5A-B). The compounds negatively correlated with *GNGT1* expression were mainly related to tyrosine kinase inhibition, DNA damage repair, PI3K/Akt/mTOR signaling, MAPK/RAF signaling, and cytotoxic chemotherapy (Figure S5C). These target classes were partially consistent with pathway enrichment results, particularly the enrichment of mTORC1-related signaling in the *GNGT1*-high group.

Molecular docking was then performed as an exploratory analysis for selected candidate compounds. The four compounds with high predicted binding affinities were ibrutinib (−9.57 kcal/mol), IU1 (−8.94 kcal/mol), taselisib (−9.81 kcal/mol), and istradefylline (−7.83 kcal/mol) (Figure S5D-G). These results suggest potential interactions between *GNGT1* and selected compounds, although direct binding and pharmacological effects require experimental validation.

Together, these findings provide hypothesis-generating evidence that *GNGT1* expression may be associated with altered drug sensitivity, but experimental validation is required to confirm its role in therapeutic response.

**Figure S5 Drug sensitivity prediction and molecular docking analysis associated with *GNGT1* expression.** (A-B) Volcano plots showing correlations between *GNGT1* expression and predicted drug IC50 values based on the CTRP and GDSC2 databases. (C) Bar plot showing the major target classes of compounds negatively correlated with *GNGT1* expression. (D-G) Molecular docking models showing the predicted interactions between *GNGT1* and four candidate compounds with high binding affinities.

**Table S1 The information of public datasets in the study**

| Number | Platform | Type | Samples | Sample source |
| --- | --- | --- | --- | --- |
|  |  |  | Control Experiment |  |
| GSE196756  GSE188900  GSE145370  GSE203115  GSE194116  GSE235537  GSE53625  GSE38129  GSE45670  GSE289185  GSE213331 | GPL24676  GPL24676  GPL24676  GPL24676  GPL16791  GPL24676  GPL18109  GPL571  GPL570  GPL24676  GPL16791 | ScRNA  ScRNA  ScRNA  ScRNA  BulkRNA  BulkRNA  Microarry  Microarry  Microarry  Microarry  Microarry | 3 3  7 1  7 7  2(R) 1(NR)  6 6  11 11  179 179  30 30  11 7(npCR)vs11(pCR)  14(pre) 15(post)  9(pre) 9(npCR)vs9(pCR) | Esophageal tissues  Esophageal tissues  Esophageal tissues  Esophageal tissues  Esophageal tissues  Esophageal tissues  Esophageal tissues  Esophageal tissues  Esophageal tissues  Esophageal tissues  Rectal tissues |
| GSE100797 | GPL11154 | Microarry | 10(R) 15(NR) | Melanoma |
| IMvigor210 | IMvigor210 | BulkRNA | 348 | Esophageal tissues |
| TCGA+GTEx | TCGA | BulkRNA | 283 94 | Esophageal tissues |

**scRNA-seq Datasets**

A total of four independent esophageal squamous cell carcinoma (ESCC) scRNA-seq datasets were enrolled in this study. All raw sequencing data were obtained from the Gene Expression Omnibus (GEO, https://www.ncbi.nlm.nih.gov/geo/), a public functional genomics data repository compliant with MIAME standards. The detailed cohort information is described below:

**Discovery cohort:** The GSE196756 and GSE188900 datasets, encompassing 10 primary ESCC tumor tissues and 4 matched adjacent normal esophageal tissues, were used to construct the single-cell transcriptomic atlas of the ESCC tumor microenvironment and identify specific biomarkers for malignant epithelial cells.

**Validation cohort 1:** The GSE145370 dataset, consisting of 7 ESCC tumor tissues and 7 matched adjacent normal tissues, was applied for independent validation of GNGT1 expression profiles and analysis of intercellular communication networks.

**Validation cohort 2:** The GSE203115 dataset, comprising tumor tissues from 3 ESCC patients treated with neoadjuvant immunochemotherapy (NACI), was utilized to explore the expression pattern and signaling features of GNGT1 during therapeutic response.

**Bulk Transcriptomic Datasets and Clinical Cohorts**

Bulk transcriptomic profiles of ESCC and corresponding complete clinical annotation data were downloaded from the GEO database and the UCSC Xena Functional Genomics Explorer (https://xenabrowser.net/). These datasets were categorized into the following groups:

**Differentially expressed gene screening cohort:** GSE194116 (6 ESCC tumor tissues vs. 6 adjacent normal tissues) and GSE235537 (11 ESCC tumor tissues vs. 11 adjacent normal tissues) were adopted for bulk-level differential expression analysis.

**Diagnostic and prognostic validation cohort:** GSE53625 (training set, 179 ESCC tumor tissues with complete overall survival and clinicopathological data); TCGA-ESCC (only ESCC samples were included, excluding esophageal adenocarcinoma, 94 tumor tissues) combined with GTEx normal esophageal tissues (283 cases), GSE23400 (53 tumor tissues vs. 53 normal tissues), and GSE38129 (30 tumor tissues vs. 30 normal tissues) served as independent external validation sets.

**Therapeutic response validation cohort:** ESCC neoadjuvant chemoradiotherapy cohorts (GSE45670: 11 normal tissues vs. 17 non-pathological complete response (npCR) vs. 11 pathological complete response (pCR); GSE289185: 14 pre-treatment vs. 15 post-treatment samples); rectal cancer neoadjuvant chemotherapy cohort GSE213331 (9 npCR vs. 9 pCR vs. 9 pre-treatment samples); advanced bladder cancer anti-PD-L1 immunotherapy cohort IMvigor210 (348 samples, downloaded via the IMvigor210CoreBiologies R package: http://research-pub.gene.com/IMvigor210CoreBiologies); advanced melanoma anti-PD-1 immunotherapy cohort GSE100797 (10 responders (R) vs. 15 non-responders (NR)).

**Table S2 Primer Sequences for qPCR**

| **Target Name** | **Primer** | |
| --- | --- | --- |
| *Actin* | F | *TCCTCCTGAGCGCAAGTACTCC* |
|  | R | *CATACTCCTGCTTGCTGATCCAC* |
| *GNGT1* | F | *ATGCCAGTAATCAATATTGAGGACC* |
|  | R | *TTATGAAATCACACAGCCTCCTTTG* |
